# Multiple skin neoplasms in subjects under 40 years of age in Goiania, Brazil

**DOI:** 10.1590/S0034-8910.2015049005777

**Published:** 2015-09-23

**Authors:** Samir Pereira, Maria Paula Curado, Ana Maria Quinteiro Ribeiro

**Affiliations:** I Serviço de Dermatologia. Instituto de Patologia Tropical e Saúde Pública. Universidade Federal de Goiás. Goiânia, GO, Brasil; II Registro de Câncer de Base Populacional de Goiânia. Goiânia, GO, Brasil; III International Prevention Research Institute. Ecculy, France

**Keywords:** Adult, Skin Neoplasms, epidemiology, Diseases Registries

## Abstract

**OBJECTIVE:**

To describe the trend for malignant skin neoplasms in subjects under 40 years of age in a region with high ultraviolet radiation indices.

**METHODS:**

A descriptive epidemiological study on melanoma and nonmelanoma skin cancers that was conducted in Goiania, Midwest Brazil, with 1,688 people under 40 years of age, between 1988 and 2009. Cases were obtained from *Registro de Câncer de Base Populacional de Goiânia* (Goiania’s Population-Based Cancer File). Frequency, trends, and incidence of cases with single and multiple lesions were analyzed; transplants and genetic skin diseases were found in cases with multiple lesions.

**RESULTS:**

Over the period, 1,995 skin cancer cases were observed to found, of which 1,524 (90.3%) cases had single lesions and 164 (9.7%) had multiple lesions. Regarding single lesions, incidence on men was observed to have risen from 2.4 to 3.1/100,000 inhabitants; it differed significantly for women, shifting from 2.3 to 5.3/100,000 (Annual percentage change – [APC] 3.0%, p = 0.006). Regarding multiple lesions, incidence on men was observed to have risen from 0.30 to 0.98/100,000 inhabitants; for women, it rose from 0.43 to 1.16/100,000 (APC 8.6%, p = 0.003). Genetic skin diseases or transplants were found to have been correlated with 10.0% of cases with multiple lesions – an average of 5.1 lesions per patient. The average was 2.5 in cases without that correlation.

**CONCLUSIONS:**

Skin cancer on women under 40 years of age has been observed to be increasing for both cases with single and multiple lesions. It is not unusual to find multiple tumors in young people – in most cases, they are not associated with genetic skin diseases or transplants. It is necessary to avoid excessive exposure to ultraviolet radiation from childhood.

## INTRODUCTION

Skin cancer is the most common malignant neoplasm in most countries. They are usually classified as nonmelanoma skin cancer (NMSC) and skin melanoma.^7^ ^,^ ^17^ The number of tumor cases has been increasing globally over the last decades.^10^ ^,^ ^19^

Ultraviolet radiation is recognized as the main risk factor for skin tumors.^3^ ^,^ ^20^ Other environmental factors which related to increased incidence of skin cancers were the ozone layer depletion and living in high altitude, low latitude areas.^22^ Among individual risk factors,^9^ ^,^ ^12^ ^,^ ^20^ ^,^ ^21^ fair skin, age above 40 years, genetic predisposition (albinism, xeroderma pigmentosum, epidermodysplasia verruciformis, and basal-cell nevus syndrome), and immunosuppression. Absent or improper skin protection during professional activities or leisure times allows for sunburns, mainly during childhood and adolescence, which leads to increased skin cancer risk.^8^ ^,^ ^14^

In Brazil, 98,420 new NMSC cases were expected for men and 83,710 cases were expected for women, which corresponds to estimated 100.75 and 82.24 rates for each 100,000 inhabitants. Regarding skin melanoma, 2,960 new NMSC cases were expected for men and 2,930 cases were expected for women, which corresponds to estimated 3.03 and 2.85 rates for each 100,000 inhabitants, according to the Instituto Nacional do Câncer (National Cancer Institute of Brazil).^a^

Skin melanoma incidence is thoroughly studied worldwide for all ages, but few studies describe NMSC rates for young people.^4^ ^,^ ^29^ In Brazil, those rates remain unknown. NMSC incidence on young populations is a reliable indicator for future risk trends, and studies have found those rates have been increasing for that age range.^4^ ^,^ ^5^ ^,^ ^2^ ^,^ ^7^

The NMSC is the most common kind of cancer, and it has low metastasis rates but significant morbidity.^7^ ^,^ ^17^ Skin melanoma is less common and is associated with high metastasis and mortality rates.^7^ ^,^ ^17^ One of the characteristics of NMSC is the high number of lesions, especially when the initial (primary) tumor is basal cell carcinoma (BCC). Some studies have been indicating that between 30.0% and 50.0% of patients who had previously been affected from BCC will have a new skin tumor in five years.^23^ ^,^ ^26^

Most population-based cancer records do not collect NMSC cases, limiting themselves to skin and mucosal melanomas. Others only record the first NMSC case, excluding simultaneous or separate multiple lesions.^13^ In Brazil, Goiania’s population-based cancer record is one of those which systematically includes NMSC cases in compliance with the international regulations for multiple primary tumors.^33^

The high number of lesions causes increased demand to the health care system, due to the need for multiple surgical interventions and long hospitalizations. Its annual cost in USA has been estimated to be over two billion dollars.^b^ In Sao Paulo, Southeastern Brazil, the annual cost for NMSC treatment has been estimated to be 37 million *reais* (corresponding to 66.6 million dollars)^c^ in 2010, a value which is 14.0% higher than the cost for melanoma.^31^ Knowing the incidence rate of multiple skin neoplasms in the young population allows establishing the risk and the adoption of preventive and educational measures in that age range. That may reduce both its risk and consequently its incidence, unburdening the public and private health care systems in skin cancer treatment.

This study intends to describe the trend for malignant skin neoplasms in subjects under 40 years of age in a region with high ultraviolet radiation indices.

## METHODS

This is a population-based epidemiological, descriptive study on skin cancer cases in Goiania’s, (GO state) metropolitan region between 1988 and 2009. Goiania is located in Brazil’s Midwestern region, at an average altitude of 749 meters and latitude -16°40’43’’. In 2013, it was observed to have high and very high ultraviolet radiation rates in the fall and in the winter, and extreme ones in the spring and in the summer, according to CPTEC (Center for Weather Forecasting and Climate Studies)/INPE (National Institute for Space Research).^d^ In 1988, Goiania’s metropolitan region was recorded to have a population of 1,007,432, of whom 80.4% people were younger than 40 years of age (392,700 men and 417,300 women). In 2009, Goiania’s population was 1,792,743, of whom 70.0% people were younger than 40 years of age (617,758 men and 636,821 women). According to the 2010 census, the population is composed of white (47.8%), brown (44.8%), black (5.7%), indigenous (0.2%), and Asian (1.7%) people.^e^

Cases were obtained from Goiania’s Population-based Cancer File (Registro de Câncer de Base Populacional de Goiânia). Variables analyzed were gender, age, diagnose date, morphology, topography, and number of lesions. Multiple skin cancer lesions are the ones in which other lesions are simultaneously observed as well the first tumor (synchronous lesions) or when other lesions appear six months after the first lesion is diagnosed (asynchronous lesions).^33^

Genetic skin diseases or transplants were found to be associated with malignant skin neoplasms and cases of multiple lesions were confirmed in the medical records of public and private health care services. Cases in which relapsing and residual tumors were observed were excluded after incisional biopsies. Patients whose records could not be accessed were invited to take part in the study and, after signing an informed consent forms, they were submitted to free medical skin examinations.

Included skin cancers were classified according to the rules from the International Classification of Diseases for Oncology, 3rd Edition (ICD-O-3). The following codes were included: 80903, 80913, 80923, 80943, 80953, 80973, and 80983, for BCC; 80513, 80703, 80713, and 80753, for squamous cell carcinoma (SCC); and 87202, 87203, 87213, 87303, 87423, 87433, 87443, 87453, and 87723, for skin melanoma.

Cases were analyzed for frequencies, genders, ages, and numbers of lesions. Patients were divided in two groups according to their number of lesions (single lesions; multiple lesions). Those groups, their morphological types and topographies were found to be associated through the use of Chi-squared and Fisher’s tests. The frequencies of tumors in cases with and without genetic skin diseases were compared for patients with multiple lesions.

Standardized incidence ratios were calculated by sex for cases with single and multiple lesions. The analysis of the incidence trend was conducted through joinpoint regression software.^18^ This software identifies the moment at which trends for change are observed (through linear analysis) and it also calculates the annual percentage change (APC) in each segment. The analysis is started by the minimum number of joinpoints, and it tests the maximum number of statistically significant joinpoints which can be added to the model. Value p < 0.05 was adopted as statistically significant.

This study was approved by the Ethics Research Committee of *Associação de Combate ao Câncer em Goiás *(ACCG– Process 30/2011).

## RESULTS

Almost 30,000 melanoma and nonmelanoma skin cancers were diagnosed between 1988 and 2009, by which 1,995 (6.9%) patients under 39 years of age were affected. Out of those, 1,524 (90.3%) patients had only one lesion and 164 (9.7%) had multiple lesions.

Among cases with single lesions, 59.7% were women and 40.3% were men – their average age was 33 years. The most frequent skin cancer was BCC (79.5%), followed by SCC (10.9%) and melanoma (9.6%) (Table 1).

**Table 1:** Distribution of skin cancer types per gender according to the number of lesions per patient. Goiania, GO, Midwestern Brazil, 1988-2009.

Morphology	Gender	Lesions						p*	RR	95%CI
		Single		Multiple		Total				
		n	%	n	%	n	%			
BCC	Male	483	39.9	187	46.5	670	41.5	0.02	0.93	0.88;0.99
	Female	729	60.1	215	53.5	944	58.5			
SCC	Male	76	45.8	32	57.1	108	48.6	0.19	0.89	0.76;1.0
	Female	90	54.2	24	42.9	114	51.4			
SM	Male	55	37.7	3	23.1	58	36.5	0.45	1	0.96;1.15
	Female	91	62.3	10	76.9	101	63.5			

BCC: basal cell carcinoma; SCC: squamous cell carcinoma; SM: skin melanoma

* Chi-square test.

Topography was defined in 92.6% of cases. The most common locations for NMSC (both BCC and SCC) were around the head and neck, followed by the trunk, upper and lower limbs (Table 2). Skin melanoma was most frequent in the trunk, followed by the limbs (Table 2).

**Table 2:** Distribution of skin cancers according to topography, gender, and number of lesions. Goiania, GO, Midwestern Brazil, 1988-2009.

Topography	BCC^a^				p	SCC^a^				p	SM^b^				p
	Single		Multiple			Single		Multiple			Single		Multiple		
	Male	Female	Male	Female		Male	Female	Male	Female		Male	Female	Male	Female	
HN	326	537	118	129	0.01	50	59	26	12	0.03	7	12	0	5	0.27
Face	293	501	95	110	0.02	44	57	23	12	0.04	5	7	0	5	0.24
Neck	22	25	22	17	0.5	4	2	3	0	0.77	0	5	0	0	–
SA	11	11	1	2	0.94	2	0	0	0	–	1	0	0	0	–
Trunk	73	100	42	44	0.38	7	12	4	2	0.41	25	26	2	1	1
Back	27	44	22	17	0.1	2	3	1	0	1	16	16	1	1	1
Thorax	45	54	19	26	0.85	2	7	3	2	0.4	8	6	1	0	1
Abdomen	1	2	1	1	0.57	3	2	0	0	0.54	1	4	0	0	–
UULL	39	42	25	39	0.35	7	11	2	9	0.44	9	13	0	3	0.28
LLLL	3	2	2	3	1	6	3	0	1	0.82	9	33	1	1	0.41
WOS	43	47	0	0	–	6	5	0	0	–	5	7	0	0	–
Total	484	728	187	215	–	76	90	32	24	–	55	91	3	10	–

BCC: basal cell carcinoma; SCC: squamous cell carcinoma; SM: skin melanoma; SA: scalp area; HN: head and neck; UULL: upper limbs; LLLL: lower limbs; WOS: with no other specification

a Chi-Squared Test.

b Fisher’s Test.

The skin cancer incidence rate in young men rose from 2.4 to 3.1 per 100,000 inhabitants between 1988 and 2009. In women, in turn, it rose from 2.3 to 5.3 per 100,000 inhabitants (Figure). The trend for incidence rose significantly in the female gender (APC = 3.0% [95%CI 0.9;5.1], p = 0.006).

**Figure:**
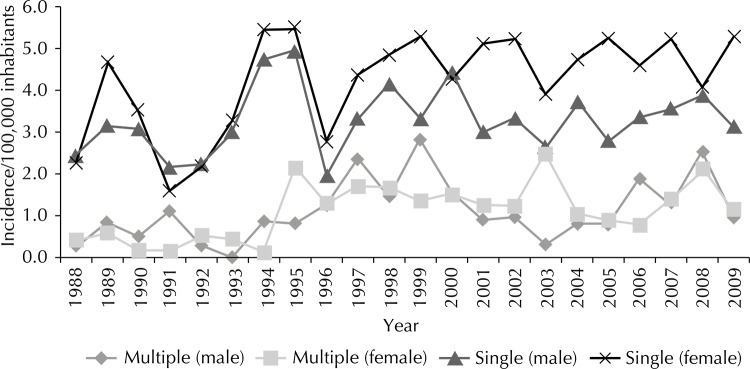
World age-standardized rates of patients under 40 years of age with single and multiple skin cancer lesions per gender. Goiania, GO, Midwestern Brazil, 1988-2009.

Among cases with multiple lesions, 55.5% were women and 44.5% were men. 471 primary neoplastic lesions were found, with an average 2.8 lesions per case, 2.7 of which being observed in women and 3.0 in men.

Regarding multiple lesions, the most frequent skin cancer was BCC (85.3%), followed by SCC (11.9%) and melanoma (2.8%) (Table 1). The BCC was most commonly observed on head-and-neck and trunk regions, and SCC was most predominant on the head and neck and on upper limbs. Melanoma was most commonly observed on head-and-neck and trunk regions (Table 2).

The incidence rate of multiple neoplasms increased from 0.30 a 0.98 per 100,000 inhabitants on males between 1988 and 2009. For women, it increased from 0.43 to 1.16 per 100,000 inhabitants (Figure). The trend for incidence in these 22 years was observed to show a significant rise in women (APC = 8.6% [95%CI 3.2;14.3], p = 0.003).

Presence of genetic skin diseases was identified in 16 patients with multiple lesions. Among those, eight were albinism cases; five of them were xeroderma pigmentosum cases; two of them were basal-cell nevus syndrome, one case was epidermodysplasia verruciformis, and one case was a kidney transplant (Table 3).

**Table 3:** Distribution of patients with multiple lesions per gender, average age at first lesion, morphological type, and lesions per patient. Goiania, GO, Midwestern Brazil, 1988-2009.

Multiple lesions	Gender		Average age at first lesion		Number of lesions						Lesions/patient	
					BCC		SCC		SM			
	Male	Female	Male	Female	n	%	n	%	n	%	Male	Female
With risk factors	10	7	30.9	26.1	64	73.6	20	23.0	3	3.4	5	5.3
Albinism	5	3	28.2	27.7	30	76.9	9	23.1	0	0	3.4	7.3
XP	2	3	30.5	42.7	8	44.4	7	3.9	3	16.7	1.7	4.3
BCNS	2	0	34	0	24	100	0	0	0	0	12	0
EPV	0	1	0	26	1	50.0	1	50.0	0	0	0	2
TX	1	0	39	0	1	25.0	3	75.0	0	0	4	0
Without these risk factors	62	84	33.9	32.3	336	88.0	36	9.4	10	2.6	2.7	2.5
All	72	91	33.5	31.8	400	85.3	56	11.9	13	2.8	3	2.7

BCC: basal cell carcinoma; SCC: squamous cell carcinoma; SM: skin melanoma; XP: xeroderma pigmentosum; BCNS: basal-cell nevus syndrome; EPV: epidermodysplasia verruciformis; TX: transplant.

f Tovo LFR, Festa Neto C, Castro CVB, Sampaio SAP. Carcinoma basocelular. Rio de Janeiro (RJ): Sociedade Brasileira de Dermatologia; 2002 [cited 2014 May 6]. (AMB Projeto Diretrizes). Available from: http://www.projetodiretrizes.org.br/projeto_diretrizes/028.pdf

Among cases with multiple lesions, without genetic skin diseases or transplants, 70.6% of patients were found to have two neoplasms; 14.4%, three; 8.2%, four; 2.7%, five; 2.0%, six; and 0.7%, above eight lesions. Out of those 65.0% were diagnosed with a second primary tumor within 6 months after the first diagnose, and 84.2% were diagnosed with it in up to three years.

## DISCUSSION

Skin cancers are the most frequents malignant neoplasms in countries with predominant Caucasian populations.^19^ Most studies include all age ranges and find high incidence of that tumor among populations of people above 40 years of age. Such behavior in younger people was described by Christenson et al^4^ and Skellettet et al.^29^ In this study, the choice was to study the incidence of skin cancer on people under 40 years of age. 1,995 lesions from NMSC and skin melanoma were found in 1,688 patients. Out of those, 9.7% had multiple lesions. Raasch et al,^25^ in a study with all age ranges, found 38.5% prevalence of multiple lesions. Therefore, a high prevalence for multiple tumors was found, which suggests they are common for patients under 40 years of age.

The incidence of malignant skin neoplasms in patients under 40 years of age was higher in women, both for single and multiple lesions. Studies investigating the incidence of skin cancer in all age ranges and genders found higher rates for incidence of single and multiple tumors in men.^7^ ^,^ ^27^ Therefore, the higher risk for women that was found by this study differs from results in the literature. That may be understood as the tropical climate in the Midwestern region of Brazil stimulates women, young people, and children to wear clothes which cover the body less, with consequent sun exposure and higher incidence of sunburns. People are also used to tanning on weekends (intense exposure over a short period). Another reason for the higher incidence on women is related to the fact they have medical appointments more frequently, which may contribute to the increased number of new cases recorded.

The incidence rates found herein for ages up to 39 years were 2 to 5 per 100,000 inhabitants. They are below the ones described for the USA, whose population has fair skin (low Fitzpatrick scale category) and increased skin cancer risk.^4^ Christenson et al,^4^ when studying the same age range in this study, found BCC incidence rates of 20.7 and 31.0 per 100,000 inhabitants between 1985 and 1989 and between 1995 and 1999 for both genders. The SCC rates went from 2.8 to 6.1 per 100,000 inhabitants in the same periods. Increased skin pigmentation enhances ultraviolet radiation protection. The Brazilian population has a highly mixed racial background, and Goiania city has 44.0% brown people, according to the 2010 census,^e^ which can explain the low rates observed.

The incidence rate oscillations with the peaks that were found in this study reflect the instability in the data which were collected by the records. Many times, lesions are submitted to treatment and the material analyzed histologically, which precludes the case from being recorded. However, such oscillations remained being observed during the whole length of the research, allowing time-related results to be safely analyzed.

The BCC was the most frequent morphological type in cases with single and multiple lesions. Women were the most affected group in both lesion types; however, the difference regarding genders was only significant in the group with single lesions. The SCC was most common in women with single lesions and in men with multiple lesions (with no significant difference). The most frequent topography in the NMSC (BCC and SCC) is the region around the head and neck, predominantly in the face, similar to what had already been described for young people.^4^ ^,^ ^29^ Those results suggest the cancer-cause role of ultraviolet radiation in the development of those neoplasms, and they reinforce the importance of prevention programs, with skin protection being included since childhood. As a matter a fact, research has been showing that sunburning is a risk factor for skin cancers in children and adolescents.^6^ ^,^ ^24^

The ratio between BCC and SCC lesion numbers was 7:1 in both groups (single and multiple lesions). The ratios between BCC and SCC were described as 3:1 and 4:1 when analyzed in all age ranges.^7^ ^,^ ^17^ In this study, BCC was found to be highly frequent as compared to SCC in young people. Christenson et al,^4^ when evaluating NMSC in the same age range as the one in this study (0 to 39 years), found the same ratio (6:1) in the USA. The BCC origin is linked to intense, intermittent ultraviolet radiation,^20^ and there is no consensus regarding its latency period in young people. The genesis of most SCC cases is related to chronic exposure to ultraviolet radiation,^20^ which is not common during childhood and adolescence. The high risk of BCC that is observed for young people as compared to the one from SCC shows the need to effectively prevent this age range. According to Deady et al,^5^ excessive exposure to ultraviolet radiation may be more observed in young people whose families have higher purchasing power, due to the traveling they do, which can lead them to intense and intermittent exposure to radiation. Such exposure leads to an earlier start of carcinogenesis, which causes these tumors to have earlier onsets. In this study, the socioeconomic factor could not be evaluated, as data regarding that were not available.

Skin melanoma was most common on women (single or multiple lesions). Different countries have different genders been shown as the most affected, and the data in this study corroborate findings regarding Brazil.^17^ ^,^ ^30^ Of the cases, 9.6% were observed to comprise single lesions and 2.8%, multiple lesions. Studies analyzing all ages found shares ranging from 3.0% to 7.0%.^7^ Probably, the group with single lesions was found to have a higher share because BCC is less frequent before the age of 40, whereas skin melanoma is common in that age range.^7^ The most frequent location in women with single lesions was in the lower limbs - in men, it was in the back, which is similar to what is found in the literature.^30^

In the group with multiple skin cancer lesions, 10.4% of cases were observed to include genetic skin diseases or transplants. In those, malignant skin neoplasms were the most frequent in men. The average age of tumor onset in cases with single lesions was 33 years for both genders. In cases with multiple lesions and genetic skin diseases, the first lesion was diagnosed between years 19 and 36, with the average age being 28 years. The number of lesions in those cases is approximately twice as big for those with no genetic diseases associated.

Patients with multiple lesions with no genetic skin diseases or transplants until the age of 40 corresponded to almost 90.0% of all cases. For them, the highest frequency of lesions was in the female gender, which is different from patients who had genetic diseases. The average number of lesions per patient was 2.5 in men and women; 15.0% of them had above three lesions. A ultraviolet radiation and sunburns may account for that increased percentage of lesions^24^ or even for the association with another yet unidentified risk factor. There is no consensus, in most countries, regarding latency periods for the onset of new lesions after the first nonmelanoma skin cancer. The British guideline suggests that BCC patients or patients with recurring neoplasms be followed up for at least three years.^32^ The German guideline recommends that all BCC patients be followed up for at least three years.^15^ In this research, 65.0% and 84.0% of second primary tumors were found to respectively have onsets in the first sixth months and in up to three years for subjects who had no genetic skin diseases or transplants. Therefore, annual and continuous dermatological supervision is required for all people who had skin cancer, especially the young patients. Special attention must also be paid during the first months after the first neoplastic skin lesion is diagnosed. In Brazil, supervision of BCC cases is recommended for long periods, with no mention to lengths.^f^

In this study, we have not evaluated patients’ professions, socioeconomic levels, or presence of HIV or HPV infections. Some studies have pointed out that HIV-infected patients^28^ and patients infected by some HPV subtypes are observed to have higher NMSC incidence rates, especially SCC.^1^ In this study, presence of HIV or HPV was not investigated due to limitations in the recording process, through which that information was not collected. Skin HPV presence is common in the skin; however, we do not have data on the prevalence of that virus in young people in Brazil. The roles for socioeconomic factors and skin cancer risk in HIV and HPV-infected people must be further investigated. Nonetheless, this study makes an important contribution as it has followed up a population for 22 years and studied the presence of skin cancers in a population of up to 39 years. The findings reinforce the need for habits to be changed, with NMSC prevention measures being adopted from early ages, by the use of hats, sunscreen, and clothes covering the limbs at times of increased risk.^2^ ^,^ ^8^ ^,^ ^14^

Educating the young population regarding the risks from excessive exposure to ultraviolet radiation is the best way to reduce the risk of nonmelanoma and melanoma skin cancers.

In this study, we observed that the incidence of skin cancer is observed to be increasing on women under 40 years of age for both cases with single and multiple lesions in Goiania, Midwestern Brazil. It is usual to find multiple skin tumors in young people (around 10.0%) – in most cases, they are not observed to be associated with genetic skin diseases or transplants. The high incidence of ultraviolet radiation and the increased exposure to it by the population during childhood and adolescence, through sunburns and clothes exposing large body areas may be one of the factors, as well as the socioeconomic levels. Excessive exposure to ultraviolet radiation is advised, from childhood, to be reduced through educational programs to parents, children, and young adults. Treated NMSC and skin melanoma cases should be continuously and systematically followed up to enable early detection of new lesions and proper treatment.

## Footnotes

a Instituto Nacional do Câncer. Câncer de pele. Brasília (DF): INCA; 2014 [cited 2015 Jun 28]. Available from: http://www.inca.gov.br/estimativa/2014/sintese-de-resultados-comentarios.asp

b National Cancer Institute. The cost of cancer [Internet]. Bethesda (MD); 2011 [cited 2013 Aug 7]. Available from: http://www.cancer.gov/aboutnci/servingpeople/cancer-statistics/costofcancer. 2011.

c Exchange Rate Conversion Factor: 1.0 dollar = 1.80 *real*, in 2010.

d DAS Customer Service Log – TUS/DAS. IUV Goiânia [internet]. Message to: Samir Pereira. October 27, 2014 [cited October 27, 2014] [1 paragraph]. Accompanied by 1 document.

e Instituto Brasileiro de Geografia Estatística. Censo Demográfico 2010. Rio de Janeiro (RJ); 2010 [cited 2012 Oct 1]. Available from: http://www.ibge.gov.br/cidadesat/topwindow.htm?1
