# Supplementary material for: Multiple skin neoplasms in subjects under 40 years of age in Goiania, Brazil
Source: Rev Saude Publica. 2015 Sep 23;49:64. doi: 10.1590/S0034-8910.2015049005777 (PMC4587818; doi:10.1590/S0034-8910.2015049005777)
Supplement: Translation

# Translation: Neoplasias múltiplas de pele em indivíduos com menos de 40 anos em Goiânia, Brasil

**Authors:** Samir Pereira, Maria Paula Curado, Ana Maria Quinteiro Ribeiro

**Affiliations:** I Serviço de Dermatologia. Instituto de Patologia Tropical e Saúde Pública. Universidade Federal de Goiás. Goiânia, GO, Brasil; II Registro de Câncer de Base Populacional de Goiânia. Goiânia, GO, Brasil; III International Prevention Research Institute. Ecculy, France

**Keywords:** Adulto, Neoplasias Cutâneas, epidemiologia, Registros de Doenças

## Abstract

**OBJETIVO:**

Descrever a tendência da incidência de neoplasias malignas de pele em indivíduos com menos de 40 anos de idade em região de altos índices de raios ultravioleta.

**MÉTODOS:**

Estudo epidemiológico descritivo de câncer de pele melanoma e não melanoma realizado em Goiânia, Brasil, com 1.688 pessoas com menos de 40 anos de idade, no período de 1988 a 2009. Os casos incidentes foram obtidos do Registro de Câncer de Base Populacional de Goiânia. Foram analisadas: frequência, tendência e incidência de casos com lesões únicas e múltiplas e, nos casos de lesões múltiplas, a presença de transplante e de doenças genéticas cutâneas.

**RESULTADOS:**

No período, ocorreram 1.995 casos de câncer de pele, dos quais 1.524 (90,3%) foram de lesão única e 164 (9,7%), lesões múltiplas. Das lesões únicas, a incidência em homens aumentou de 2,4 para 3,1/100.000 habitantes; em mulheres, diferiu significativamente, passando de 2,3 para 5,3/100.000 (*Annual percentage change* – [APC] 3,0%, p = 0,006). A incidência de lesões múltiplas em homens aumentou de 0,30 para 0,98/100.000 habitantes; nas mulheres, o aumento foi de 0,43 para 1,16/100.000 (APC 8,6%, p = 0,003). Encontrou-se associação com doença genética cutânea ou transplante em 10,0% dos casos com lesões múltiplas, com média de 5,1 lesões por paciente. Nos casos sem associação essa média foi de 2,5.

**CONCLUSÕES:**

A incidência de câncer de pele em mulheres com menos de 40 anos está aumentando tanto com lesões únicas, quanto com múltiplas. A ocorrência de tumores múltiplos em jovens não é raro e na maioria dos casos não está associado à doença genética cutânea ou transplante. É necessário prevenir a exposição excessiva à radiação ultravioleta desde a infância.

## INTRODUÇÃO

O câncer de pele é a neoplasia maligna mais comum na maioria dos países. Habitualmente, é classificado em câncer de pele não melanoma (CPNM) e melanoma cutâneo.^7^ ^,^ ^17^ A incidência destes tumores aumentou globalmente nas últimas décadas.^10^ ^,^ ^19^

A radiação ultravioleta solar é reconhecida como o principal fator de risco para tumores cutâneos.^3^ ^,^ ^20^ Outros fatores ambientais relacionados à maior incidência de câncer de pele são a redução da camada de ozônio e viver em regiões de alta altitude e baixa latitude.^22^ Entre os fatores de risco individuais,^9^ ^,^ ^12^ ^,^ ^20^ ^,^ ^21^ encontram-se pele clara, idade acima de 40 anos, predisposição genética (albinismo, xeroderma pigmentoso, epidermodisplasia verruciforme e síndrome do nevo basocelular) e imunossupressão. A ausência ou a inadequada proteção da pele nas atividades laborais e nos momentos de lazer propiciam queimaduras solares, principalmente na infância e adolescência, o que leva ao aumento do risco de câncer de pele.^8^ ^,^ ^14^

No Brasil, o número de casos novos de CPNM esperados para o ano de 2014 foi de 98.420 em homens e 83.710 em mulheres, o que corresponde às taxas estimadas de 100,75 e 82,24 a cada 100.000. Para melanoma cutâneo, foi de 2.960 em homens e 2.930 em mulheres, o que corresponde às taxas de 3,03 e 2,85 por 100.000, segundo o Instituto Nacional do Câncer.^a^

A incidência do melanoma cutâneo é bem estudada globalmente para todas as idades, mas poucos estudos descrevem as taxas de CPNM em jovens.^4^ ^,^ ^29^ No Brasil, essas incidências permanecem desconhecidas. A incidência do CPNM em populações jovens é um indicador confiável de tendências de risco futuras e estudos têm demonstrado aumento das taxas nesta faixa etária.^4^ ^,^ ^5^ ^,^ ^27^

O CPNM é o câncer mais incidente e possui baixa taxa de metástases, mas significativa morbidade.^7^ ^,^ ^17^ O melanoma cutâneo é menos comum e está associado a altas taxas de metástases e mortalidade.^7^ ^,^ ^17^ Uma das características do CPNM é a multiplicidade de lesões, especialmente quando o tumor inicial (primário) é o carcinoma basocelular (CBC). Algumas pesquisas têm indicado que entre 30,0% e 50,0% dos pacientes com CBC prévio terão um novo tumor de pele em cinco anos.^23^ ^,^ ^26^

A maioria dos registros de câncer de base populacional não coleta casos de CPNM, limitando-se aos melanomas de pele e mucosa. Outros registram apenas o primeiro caso de CPNM, excluindo as lesões múltiplas sincrônicas ou assincrônicas.^13^ No Brasil, o Registro de Câncer de Base Populacional de Goiânia é um dos que inclui sistematicamente os casos de CPNM seguindo as regras internacionais de tumores primários múltiplos.^33^

A multiplicidade de lesões causa maior demanda do sistema de saúde devido à necessidade de múltiplas intervenções cirúrgicas e longo seguimento. O custo anual nos EUA foi estimado em mais de dois bilhões de dólares.^b^ Em São Paulo, o custo anual do tratamento do CPNM foi estimado em 37 milhões de reais (equivalente a 66,6 milhões de dólares)^c^ em 2010, valor 14,0% maior que o custo para melanoma.^31^ Conhecer a incidência das neoplasias múltiplas de pele na população jovem possibilita estabelecer o risco e a adoção de medidas preventivas e educativas nesta faixa etária. Isso poderá reduzir o risco e, consequentemente, a incidência, desonerando os sistemas públicos e privados de saúde no tratamento do câncer de pele.

O objetivo deste estudo foi descrever a tendência da incidência de neoplasias malignas de pele em indivíduos com menos de 40 anos de idade em região de altos índices de raios ultravioleta.

## MÉTODOS

Trata-se de estudo epidemiológico descritivo de base populacional dos casos incidentes de câncer de pele na região metropolitana de Goiânia, GO, no período de 1988 a 2009. Goiânia está localizada na região Centro-Oeste do Brasil, a uma altitude média de 749 metros e latitude de -16°40’43’’. Em 2013, apresentou índices ultravioleta alto e muito alto no outono e inverno, e extremo na primavera e no verão, segundo o CPTEC/INPE.^d^ No ano de 1988, a região metropolitana de Goiânia apresentava população de 1.007.432, sendo 80,4% com menos de 40 anos de idade (392.700 homens e 417.300 mulheres). Em 2009, a população era de 1.792.743, sendo 70,0% com idade inferior a 40 anos (617.758 homens e 636.821 mulheres). Segundo o censo 2010, a população é composta por brancos (47,8%), pardos (44,8%), pretos (5,7%), indígenas (0,2%) e amarelos (1,7%).^e^

Os casos incidentes foram obtidos do Registro de Câncer de Base Populacional de Goiânia. As variáveis analisadas foram sexo, idade, data do diagnóstico, morfologia, topografia e número de lesões. São consideradas lesões múltiplas de câncer de pele a presença de uma segunda ou mais lesões concomitantes ao primeiro tumor (lesões sincrônicas) ou mais de seis meses após o diagnóstico da primeira lesão (lesões assincrônicas).^33^

Foram verificados nos prontuários médicos de hospitais públicos e serviços privados a presença de doença genética cutânea ou transplantes associados à ocorrência de neoplasias malignas da pele e confirmados os casos registrados de lesões múltiplas. Casos com recidivas e tumores residuais após biópsia incisional foram excluídos. Pacientes com prontuários inacessíveis foram convidados a participar da pesquisa e, após assinatura do termo de consentimento livre e esclarecido, foram submetidos à consulta médica dermatológica gratuita.

Os cânceres de pele incluídos foram classificados conforme a Classificação Internacional de Doenças para Oncologia 3ª edição (CIDO-3). Foram incluídos os códigos para CBC: 80903, 80913, 80923, 80943, 80953, 80973, 80983; carcinoma espinocelular (CEC): 80513, 80703, 80713, 80753; e melanoma da pele: 87202, 87203, 87213, 87303, 87423, 87433, 87443, 87453, 87723.

Os casos incidentes foram analisados quanto à frequência, segundo sexo, idade e número de lesões. Os pacientes foram divididos em dois grupos segundo número de lesões (lesões únicas; lesões múltiplas). Verificou-se associação entre esses grupos e os tipos morfológicos e as topografias, utilizando os testes Qui-quadrado e de Fisher. As frequências de tumores nos casos com e sem doença genética cutânea foram comparadas nos portadores de lesões múltiplas.

Foi calculada a taxa de incidência padronizada pelo sexo dos casos com lesões únicas e múltiplas. A análise da tendência de incidência foi realizada usando o programa de regressão *joinpoint*.^18^ Esse programa identifica o momento no qual as tendências de mudança ocorrem, por análise de regressão linear, e calcula a porcentagem anual de mudança, pelo *annual percentage change *(APC) em cada segmento. A análise inicia pelo número mínimo de *jointpoints *e testa o máximo de pontos agregados (*jointpoint*) que são estatisticamente significativos e podem ser adicionados no modelo. Foi adotado como significância estatística o valor de p < 0,05.

Este estudo foi aprovado pelo Comitê de Ética em Pesquisa (CEP) da Associação de Combate ao Câncer em Goiás (ACCG – Processo 30/2011).

## RESULTADOS

No período de 1988 a 2009, foram diagnosticados 28.982 casos de câncer de pele melanoma e não melanoma, dos quais 1.995 (6,9%) casos ocorreram em 1.688 indivíduos com até 39 anos. Desses, 1.524 (90,3%) pacientes tiveram uma lesão e 164 (9,7%) tiveram lesões múltiplas.

Dos casos com lesões únicas, 59,7% eram mulheres e 40,3% homens, com média de idade de 33 anos. O câncer de pele mais frequente foi o CBC (79,5%), seguido pelo CEC (10,9%) e melanoma (9,6%) (Tabela 1).

**Tabela 1:** Distribuição dos tipos de câncer de pele de acordo com o sexo pelo número de lesões por paciente. Goiânia, GO, Brasil, 1988-2009.

Morfologia	Sexo	Lesões						p*	RR	IC95%
		Únicas		Múltiplas		Total				
		n	%	n	%	n	%			
CBC	Masc	483	39,9	187	46,5	670	41,5	0,02	0,93	0,88;0,99
	Fem	729	60,1	215	53,5	944	58,5			
CEC	Masc	76	45,8	32	57,1	108	48,6	0,19	0,89	0,76;1,0
	Fem	90	54,2	24	42,9	114	51,4			
MC	Masc	55	37,7	3	23,1	58	36,5	0,45	1	0,96;1,15
	Fem	91	62,3	10	76,9	101	63,5			

CBC: carcinoma basocelular; CEC: carcinoma espinocelular; MC: melanoma cutâneo; Masc: masculino; Fem: feminino

* Teste Qui-quadrado.

Foi definida a topografia em 92,6% dos casos. A localização mais comum dos CPNM, tanto do CBC quanto do CEC, foi na região de cabeça e pescoço, seguido pelo tronco, membros superiores e inferiores (Tabela 2). O melanoma cutâneo foi mais frequente no tronco, seguido pelos membros (Tabela 2).

**Tabela 2:** Distribuição dos cânceres de pele segundo topografia, sexo e número de lesões. Goiânia, GO, Brasil, 1988-2009.

Topografia	CBC^a^				p	CEC^a^				p	MC^b^				p
	Únicas		Múltiplas			Únicas		Múltiplas			Únicas		Múltiplas		
	Masc	Fem	Masc	Fem		Masc	Fem	Masc	Fem		Masc	Fem	Masc	Fem	
CP	326	537	118	129	0,01	50	59	26	12	0,03	7	12	0	5	0,27
Face	293	501	95	110	0,02	44	57	23	12	0,04	5	7	0	5	0,24
Pescoço	22	25	22	17	0,5	4	2	3	0	0,77	0	5	0	0	–
CC	11	11	1	2	0,94	2	0	0	0	–	1	0	0	0	–
Tronco	73	100	42	44	0,38	7	12	4	2	0,41	25	26	2	1	1
Dorso	27	44	22	17	0,1	2	3	1	0	1	16	16	1	1	1
Tórax	45	54	19	26	0,85	2	7	3	2	0,4	8	6	1	0	1
Abdome	1	2	1	1	0,57	3	2	0	0	0,54	1	4	0	0	–
MMSS	39	42	25	39	0,35	7	11	2	9	0,44	9	13	0	3	0,28
MMII	3	2	2	3	1	6	3	0	1	0,82	9	33	1	1	0,41
SOE	43	47	0	0	–	6	5	0	0	–	5	7	0	0	–
Total	484	728	187	215	–	76	90	32	24	–	55	91	3	10	–

CBC: carcinoma basocelular; CEC: carcinoma espinocelular; MC: melanoma cutâneo; Masc: masculino; Fem: feminino; CC: couro cabeludo; CP: cabeça e pescoço; MMSS: membros superiores; MMII: membros inferiores; SOE: sem outra especificação

a Teste Qui-quadrado.

b Teste de Fisher.

A taxa de incidência de câncer de pele em homens jovens aumentou de 2,4 para 3,1 por 100.000 habitantes de 1988 para 2009, enquanto nas mulheres aumentou de 2,3 para 5,3 por 100.000 habitantes (Figura). A tendência da incidência aumentou significativamente no sexo feminino (APC = 3,0% [IC95% 0,9;5,1], p = 0,006).

**Figura:** Taxa de incidência padronizada por idade de pacientes com lesões únicas e múltiplas de câncer de pele pelo sexo em indivíduos com menos de 40 anos. Goiânia, GO, Brasil, 1988-2009.

Dos casos com lesões múltiplas, 55,5% eram mulheres e 44,5% homens. Foram verificadas 471 lesões neoplásicas primárias, com média de 2,8 lesões por caso, sendo 2,7 no sexo feminino e 3,0 no masculino.

Para as lesões múltiplas, o câncer de pele mais frequente foi o CBC (85,3%), seguido pelo CEC (11,9%) e melanoma (2,8%) (Tabela 1). O CBC foi mais comum na região da cabeça e pescoço e no tronco. O CEC predominou na cabeça e pescoço e nos membros superiores. O melanoma foi mais frequente na cabeça e pescoço e no tronco (Tabela 2).

A taxa de incidência de neoplasias múltiplas aumentou, no sexo masculino, de 0,30 a 0,98 por 100.000 habitantes entre 1988 e 2009. No sexo feminino, o aumento foi de 0,43 para 1,16 por 100.000 habitantes (Figura). A tendência da incidência nestes 22 anos evidenciou aumento significativo nas mulheres (APC = 8,6% [IC95% 3,2;14,3], p = 0,003).

A presença de doença genética cutânea foi identificada em 16 pacientes com lesões múltiplas, dos quais: oito eram casos de albinismo; cinco, de xeroderma pigmentoso; dois, de síndrome do nevo basocelular, um de epidemordisplasia verruciforme, e um caso de transplante renal (Tabela 3).

**Tabela 3:** Distribuição dos pacientes com lesões múltiplas segundo sexo, média de idade da primeira lesão, tipo morfológico e lesões por paciente. Goiânia, GO, Brasil, 1988-2009.

Lesões múltiplas	Sexo		Idade média à primeira lesão		Número de lesões						Lesões/paciente	
					CBC		CEC		MC			
	Masc	Fem	Masc	Fem	n	%	n	%	n	%	Masc	Fem
Com fatores de risco	10	7	30,9	26,1	64	73,6	20	23,0	3	3,4	5	5,3
Albinismo	5	3	28,2	27,7	30	76,9	9	23,1	0	0	3,4	7,3
XP	2	3	30,5	42,7	8	44,4	7	3,9	3	16,7	1,7	4,3
SNB	2	0	34	0	24	100	0	0	0	0	12	0
EPD	0	1	0	26	1	50,0	1	50,0	0	0	0	2
TX	1	0	39	0	1	25,0	3	75,0	0	0	4	0
Sem esses fatores de risco	62	84	33,9	32,3	336	88,0	36	9,4	10	2,6	2,7	2,5
Todos	72	91	33,5	31,8	400	85,3	56	11,9	13	2,8	3	2,7

CBC: carcinoma basocelular; CEC: carcinoma espinocelular; MC: melanoma cutâneo; Masc: masculino; Fem: feminino; XP: xeroderma pigmentoso; SNB: síndrome do nevobasocelular; EPV: epidermiodisplasia verruciforme; TX: transplante

f Tovo LFR, Festa Neto C, Castro CVB, Sampaio SAP. Carcinoma basocelular. Rio de Janeiro: Sociedade Brasileira de Dermatologia; 2002 [citado 2014 mai 6]. (AMB Projeto Diretrizes). Disponível em: http://www.projetodiretrizes.org.br/projeto_diretrizes/028.pdf

Dos casos com lesões múltiplas, sem doença genética cutânea ou transplante, 70,6% apresentava duas neoplasias; 14,4%, três; 8,2%, quatro; 2,7%, cinco; 2,0%, seis; e 0,7%, acima de oito lesões. Desses casos, 65,0% foi diagnosticado com segundo tumor primário nos primeiros seis meses após o primeiro diagnóstico e 84,2% recebeu esse diagnóstico em até três anos.

## DISCUSSÃO

O câncer de pele é a neoplasia maligna mais frequente nos países com população predominantemente caucasiana.^19^ A maioria das pesquisas aborda todas as faixas etárias, evidenciando altas incidências deste tumor na população com mais de 40 anos. O comportamento em pessoas mais jovens foi descrito por Christenson et al^4^ e Skellettet al.^29^ Na presente pesquisa, optou-se por estudar a ocorrência do câncer de pele em pessoas com menos de 40 anos. Foram verificadas 1.995 lesões incidentes de CPNM e melanoma cutâneo em 1.688 pacientes. Destes, 9,7% tiveram lesões múltiplas. Raasch et al,^25^ em estudo de todas as faixas etárias, observaram 38,5% de lesões múltiplas. Identificou-se, portanto, alta prevalência de tumores múltiplos, o que sugere ser comum em pacientes com menos de 40 anos de idade.

A incidência das neoplasias malignas da pele em pessoas com menos de 40 anos foi mais alta nas mulheres tanto para lesões únicas como múltiplas. Estudos que investigam a incidência de câncer de pele em todas as faixas etárias e sexo observaram maiores taxas de incidência de tumores únicos e múltiplos em homens.^7^ ^,^ ^27^ Portanto, o maior risco em relação ao sexo feminino encontrado no presente estudo difere da literatura. Isso pode ser compreendido por que o clima tropical da região Centro-Oeste do Brasil estimula as mulheres jovens e crianças a usarem roupas com menor cobertura do corpo, com exposição ao sol diuturna e maior incidência de queimaduras solares, além do hábito de bronzear-se nos finais de semana (exposição intensa em período curto). Outra razão da maior incidência em mulheres está ligada ao acesso ao diagnóstico e procura dos serviços médicos com maior frequência, o que pode contribuir para o aumento do número de casos novos registrados.

As taxas de incidência aqui encontradas para idade até 39 anos foram de 2 a 5 por 100.000 habitantes, estando aquém das descritas nos EUA, cuja população apresenta pele clara (fototipo baixo) e maior risco para câncer cutâneo.^4^ Christenson et al,^4^ ao estudarem a mesma faixa etária desta pesquisa, encontraram taxas de incidência de CBC de 20,7 e 31,0 por 100.000 habitantes nos períodos de 1985 a 1989 e de 1995 a 1999 para ambos os sexos. As taxas de CEC passaram de 2,8 a 6,1 por 100.000 habitantes, nos mesmos períodos. Maior pigmentação na pele aumenta a proteção contra radiação ultravioleta. A população brasileira apresenta grande miscigenação e a cidade de Goiânia conta com 44,0% de pardos, segundo censo 2010,^e^ o que pode explicar as baixas taxas observadas.

As oscilações das taxas de incidência com picos encontradas neste estudo refletem a instabilidade dos dados na coleta pelo registro. Muitas vezes, a lesão é submetida a tratamento e o material não é enviado para exame histológico, o que impede o registro do caso. Entretanto, estas oscilações mantiveram-se durante todo o período da pesquisa, permitindo que os resultados temporais fossem analisados com segurança.

O CBC foi o tipo morfológico mais frequente nos casos com lesões únicas e múltiplas. As mulheres foram as mais acometidas em ambos os tipos de lesões; porém, a diferença segundo sexo foi significativa apenas no grupo de lesões únicas. Já o CEC foi mais comum em mulheres com lesões únicas e nos homens com lesões múltiplas (sem diferença significativa). A topografia mais frequente dos CPNM (CBC e CEC) foi a região da cabeça e pescoço, com predomínio da face, semelhante ao descrito previamente para jovens.^4^ ^,^ ^29^ Esses resultados sugerem o papel carcinogênico da radiação ultravioleta no desenvolvimento dessas neoplasias e reforçam a importância dos programas de prevenção, com a proteção da pele desde a infância. De fato, pesquisas têm mostrado que a queimadura solar em crianças e adolescentes é um dos fatores de risco para o câncer de pele no jovem.^6^ ^,^ ^24^

A proporção do número de lesões de CBC e CEC foi de 7:1 nos dois grupos (lesões únicas e múltiplas). A razão entre CBC e CEC foi descrita como 3:1 e 4:1 quando analisada em todas as faixas etárias.^7^ ^,^ ^17^ Neste estudo, constatou-se alta frequência de CBC em relação aos CEC em jovens. Christenson et al,^4^ ao analisarem CPNM na mesma faixa etária avaliada neste estudo (0 a 39 anos), nos EUA, também encontraram alta proporção (6:1). A origem do CBC está associada à radiação ultravioleta intensa e intermitente,^20^ e não há consenso quanto ao tempo de latência em jovens. A gênese da maioria dos casos de CEC está relacionada com a exposição crônica à radiação ultravioleta,^20^ que não é comum na infância e adolescência. O alto risco observado de CBC em jovens, em relação ao CEC, denuncia a necessidade de prevenção efetiva nessa faixa etária. Segundo Deady et al,^5^ a exposição excessiva à radiação ultravioleta pode ser maior em jovens com família de maior poder aquisitivo, pelas viagens que lhes são proporcionadas e que podem levá-los a exposições intensas e intermitentes à radiação. Tais exposições iniciam o processo de carcinogênese mais cedo, antecipando o aparecimento desses tumores. Neste estudo, não foi possível avaliar o fator socioeconômico, devido à indisponibilidade desses dados.

O melanoma cutâneo foi mais comum nas mulheres (lesões únicas ou múltiplas). A maior frequência do melanoma cutâneo em relação ao sexo varia entre os países, estando estes dados de acordo com os encontrados no Brasil.^17^ ^,^ ^30^ Observou-se que 9,6% dos casos eram de lesões únicas e 2,8%, de lesões múltiplas. Estudos que analisam todas as idades encontram proporção que varia entre 3,0% a 7,0%.^7^ Provavelmente, o grupo com lesões únicas apresentou maior proporção porque o CBC é menos frequente antes dos 40 anos, enquanto o melanoma cutâneo é comum nesta faixa etária.^7^ A localização mais frequente em mulheres com lesões únicas foi nos membros inferiores e em homens no dorso, semelhante ao encontrado na literatura.^30^

No grupo com lesões múltiplas de câncer de pele, foi observado que 10,4% dos casos apresentavam doença genética cutânea ou transplante. Nesses, as neoplasias malignas cutâneas foram mais frequentes em homens. A média da idade de aparecimento do tumor nos casos de lesões únicas foi de 33 anos em ambos os sexos. Nos casos com lesões múltiplas com presença de doença genética cutânea, a primeira lesão foi diagnosticada entre os 19 e 36 anos, com média de 28 anos. O número de lesões nesses casos é cerca de duas vezes maior do que naqueles sem doença genética associada.

Os pacientes com lesões múltiplas sem doença genética cutânea ou transplante até os 40 anos de idade corresponderam a quase 90,0% dos casos. Neles, a maior frequência de lesões foi no sexo feminino, diferente dos que possuíam a associação genética. A média de lesões por paciente foi de 2,5 em homens e mulheres, sendo que 15,0% apresentaram acima de três lesões. A radiação ultravioleta e as queimaduras solares poderiam explicar esse percentual maior de lesões^24^ ou até mesmo a associação com outro fator de risco ainda não identificado. Não há consenso quanto ao tempo de seguimento para detecção de novas lesões após o primeiro câncer de pele não melanoma na maioria dos países. A diretriz britânica sugere acompanhamento por, pelo menos, três anos para pacientes com CBC ou tratados por neoplasia recorrente.^32^ A diretriz alemã recomenda seguimento anual para todos os pacientes com CBC por, pelo menos, três anos.^15^ Nesta pesquisa, verificou-se que 65,0% e 84,0% dos segundos tumores primários de pele ocorreram respectivamente nos primeiros seis meses e até três anos em indivíduos sem doença genética cutânea ou transplante. Portanto, é necessário o acompanhamento dermatológico anual e contínuo de todas as pessoas que tiveram câncer de pele, principalmente dos jovens. Deve-se ainda dar atenção especial durante os seis primeiros meses após o diagnóstico da primeira lesão neoplásica de pele. No Brasil, recomenda-se o seguimento dos casos de CBC por longo período, sem especificar o tempo.^f^

Neste estudo, não avaliamos a profissão dos pacientes, o nível socioeconômico e a ocorrência de infecção por HIV e pelo HPV. Alguns trabalhos têm apontado que pacientes infectados pelo HIV^28^ e com alguns subtipos de HPV apresentam maior incidência de CPNM, especialmente de CEC.^1^ No presente estudo, não foi investigada a presença de HIV e HPV, por limitação da própria atividade do registro, que não coleta essa informação. A presença do HPV cutâneo é comum na pele, porém não temos dados da prevalência deste vírus nas pessoas jovens no Brasil. O papel do fator socioeconômico e o risco do câncer de pele em pessoas infectadas por HIV e HPV precisam ser melhor investigados. No entanto, o presente estudo traz importante contribuição por ser de base populacional de 22 anos ininterruptos e por estudar a ocorrência de câncer de pele em uma população de até 39 anos. Os achados reforçam a necessidade de mudança de hábitos, com adoção desde a infância de práticas de prevenção do CPNM, pelo uso de roupas protetoras dos membros, chapéu e filtro protetor solar em horários de maior risco.^2^ ^,^ ^8^ ^,^ ^14^

A educação da população jovem sobre o risco da exposição excessiva à radiação ultravioleta é a melhor forma de reduzir o risco para o câncer de pele não melanoma e o melanoma.

Neste estudo, observamos que a incidência de câncer de pele está aumentando em mulheres com menos de 40 anos de idade em Goiânia, tanto para lesões únicas, quanto lesões múltiplas no Centro-Oeste do Brasil. A ocorrência de tumores múltiplos de pele em jovens é comum (cerca de 10,0%) e, na maioria dos casos, não está associada à doença genética cutânea ou transplante. Acredita-se que a alta incidência da radiação ultravioleta, associada à maior exposição da sua população na infância e adolescência com queimaduras solares e o uso de roupas que expõem grande parte do corpo, pode ser um dos fatores, assim como o nível socioeconômico. Recomenda-se a redução da exposição excessiva à radiação ultravioleta desde a infância por meio de programas educativos para os pais, crianças e adultos jovens. Os casos tratados de CPNM e melanoma cutâneo devem ter seguimento contínuo e sistemático para a detecção precoce de novas lesões e tratamento adequado.

## Footnotes

a Instituto Nacional do Câncer. Câncer de pele. Brasília (DF): INCA; 2014 [citado 2015 jun 28]. Disponível em: http://www.inca.gov.br/estimativa/2014/sintese-de-resultados-comentarios.asp

b National Cancer Institute. The cost of cancer [Internet]. Bethesda (MD); 2011 [citado 2013 ago 7]. Disponível em: http://www.cancer.gov/aboutnci/servingpeople/cancer-statistics/costofcancer. 2011.

c Fator de conversão: 1,0 dólar = 1,80 real, em 2010.

d Atendimento ao Usuário DAS – TUS/DAS. IUV Goiânia [internet]. Mensagem para: Samir Pereira. 27 out 2014 [citado 2014 out 27] [1 parágrafo]. Acompanhado por 1 documento.

e Instituto Brasileiro de Geografia Estatística. Censo Demográfico 2010. Rio de Janeiro (RJ); 2010 [citado 2012 out 1]. Disponível em: http://www.ibge.gov.br/cidadesat/topwindow.htm?1
